# Evaluation of Inpatient Surveillance After High-Energy Accident Without Apparent Serious Injury: Retrospective Analysis of Necessary Interventions and Their Predictors

**DOI:** 10.3390/jcm15082831

**Published:** 2026-04-08

**Authors:** Andreas Gather, Alexandra Braun, Matthias K. Jung von Landenberg, Paul Alfred Gruetzner, Philipp Raisch

**Affiliations:** 1 Department of Interdisciplinary Emergency and Acute Medicine (IRN), BG Klinik Ludwigshafen, Ludwig-Guttmann-Str. 13, 67071 Ludwigshafen, Germany; andreas.gather@bgu-ludwigshafen.de; 2 Department for Orthopaedics and Trauma Surgery at Heidelberg University, BG Klinik Ludwigshafen, Ludwig-Guttmann-Str. 13, 67071 Ludwigshafen, Germany; alexandra.braun@bgu-ludwigshafen.de (A.B.); matthias.jung@bgu-ludwigshafen.de (M.K.J.v.L.); paul.gruetzner@bgu-ludwigshafen.de (P.A.G.)

**Keywords:** high-energy trauma, inpatient surveillance, missed injury, tertiary survey, trauma diagnostics

## Abstract

**Background/Objectives:** Patients involved in high-energy accidents (HEAs) are frequently admitted for inpatient surveillance despite normal clinical examination and imaging, although the yield of this practice is uncertain. This study evaluated the frequency and nature of clinical events, interventions during surveillance and missed injuries in such low-risk patients and explored potential predictors. **Methods:** Retrospective study at a Level I trauma center including patients ≥18 years admitted between January 2022 and September 2023 solely due to HEA mechanism, without apparent injury requiring inpatient treatment. Baseline characteristics, clinical presentation, imaging findings, and laboratory values were extracted. Outcomes included additional diagnostics, new diagnoses, therapeutic interventions, and missed injuries. Patients with eventful and uneventful stays were compared using univariate statistical tests. **Results:** Among 363 included patients, 86.0% experienced an uneventful stay. Fifty-one patients (14.0%) had an eventful stay, most commonly requiring additional radiological examinations (8.5%) or blood tests (6.9%). New diagnoses occurred in 6.6%, and 6.1% received additional therapeutic interventions. Missed injuries were detected in 3.9%, including two potentially life-threatening injuries (0.6%). No robust predictors for missed injuries were identified. Established predictors of missed injuries from broader trauma populations were absent in this selected low-risk cohort. However, individuals after bicycle accidents were significantly more likely to experience any event during their stay (*p* = 0.009). **Conclusions:** Inpatient surveillance of patients without apparent injury after HEAs has a low overall yield but occasionally identifies clinically significant conditions. As no reliable predictors for adverse events were found, selective admission remains challenging. Hybrid models combining short-term observation with structured outpatient reassessment may represent a resource-efficient alternative for low-risk patients.

## 1. Introduction

High-energy accidents (HEAs) remain a concern in emergency medicine because they are associated with complex injury patterns and a recognized risk of occult injury. Major trauma care frameworks, such as the Advanced Trauma Life Support^®^ (ATLS^®^) program [1], emphasize mechanism of injury as one of the key triggers for intensified diagnostic evaluation. Consequently, patients involved in HEAs are frequently admitted for inpatient observation even when the initial clinical examination and imaging studies are unremarkable.

Despite systematic evaluation, including structured primary and secondary surveys and the widespread use of whole-body CT, some injuries remain occult during the initial assessment. Several authors have described missed injuries or delayed diagnoses occurring hours after HEAs, particularly involving the abdomen, thorax, or musculoskeletal system [2,3]. This residual diagnostic uncertainty has historically justified conservative management and routine inpatient surveillance based solely on mechanism.

However, an emerging body of evidence suggests that the clinical yield of such surveillance is low in patients without apparent injury, with missed injury rates of 0.0–8.0% [4,5,6] and serious initially missed injuries in only 0.5% [6]. Collectively, these findings raise questions regarding the justification of mandatory inpatient surveillance, particularly in overstretched healthcare systems increasingly shifting toward ambulatory care. Likewise, the current German S3 polytrauma guideline emphasizes that trauma team activation and intensive imaging should primarily be guided by physiological instability and defined injury patterns, with accident mechanism relegated to a secondary, moderate-risk criterion (fall > 3 m, motor vehicle accident (MVA) with ejection of patient). For whole-body CT, only a few clearly defined mechanisms (fall > 4 m, thoracic or abdominal entrapment) are retained as one of several triggers, reflecting a growing emphasis on multimodal clinical criteria beyond mechanism alone [7].

Attempts to identify predictors of missed injuries or other clinically relevant in-hospital events have produced inconsistent results. Established risk factors, such as high injury severity, polytrauma, altered mental status, or otherwise impaired clinical assessability, have been identified in general trauma populations [2] but are largely absent in the subset of patients admitted solely because of HEAs. As a result, no reliable tool currently exists to identify the small proportion of patients who may still be at risk for delayed clinical deterioration. Furthermore, most previous studies have focused narrowly on missed injuries as the primary endpoint [2,4,5,6], while other potentially meaningful in-hospital events, such as additional diagnostics, therapeutic interventions, or symptom-driven reassessment, have rarely been analyzed in detail.

In light of these uncertainties, a more comprehensive evaluation of the clinical yield of inpatient surveillance in patients presenting after HEAs without apparent injury is warranted. The aim of this study was therefore to assess the frequency and nature of events, interventions, and new diagnoses during inpatient observation in this low-risk population, and to explore potential predictors for eventful hospital stays or missed injuries.

## 2. Materials and Methods

This study was conducted at a Level I trauma center in Germany. It received approval from the local ethics board (Ethics Committee of the State Medical Association of Rhineland-Palatinate, Mainz, Germany; application 2025-18513; approval date: 15 December 2025) and is reported in accordance with the STROBE statement [8].

Retrospectively, records of patients ≥18 years admitted for inpatient treatment via the hospital’s emergency room (ER) between January 2022 and September 2023 were analyzed. Patients were included if the decision for inpatient treatment and surveillance was based solely on the mechanism of a HEA. At the study site, the classification of HEAs during the inclusion period was primarily oriented along the mechanism-based B-criteria of earlier versions of the German S3 guideline (e.g., high-speed collisions, vehicle rollover, ejection from the vehicle, pedestrian impact, motorcycle accidents, and falls from height). However, the final decision for admission is made on an individual basis by the treating trauma surgeon and may incorporate additional clinical judgment.

Thus, patients were excluded if they presented with an injury or condition requiring inpatient treatment or surveillance, if they were admitted for inpatient medication or because of deficits in domestic care or support. Patients were also excluded if they had only fallen from standing height, which was sometimes the case in elderly patients. Details on inclusion and exclusion criteria are presented in Table 1.

Patients admitted to the ER after HEA are routinely assessed based on ATLS^®^ principles, involving a structured primary and secondary survey [1]. Routinely, inpatient surveillance is recommended even if no serious injury is detected and, if not declined by the patient, patients are admitted for at least 24 h of clinical surveillance. Within this time, a tertiary trauma survey is performed. Additional diagnostic measures (e.g., radiographic, blood tests) are done, diagnoses made and therapies indicated, if necessary.

For the included patients, the following baseline variables were extracted from medical records:

- Demographic: Age, gender, preexisting conditions, resulting age-adjusted Charlson Comorbidity Index (CCI) [9] and American Society of Anesthesiologists (ASA) classification, direct oral anticoagulants (DOAKs) and anti-platelet medication (APM);
- Injuries: Injuries apparent upon admission based on Abbreviated Injury Scale (AIS) [10] nomenclature and resulting Injury Severity Score (ISS) [11];
- Type of accident: Car accident, bicycle, motorcycle accident, fall lower and greater than 3 m;
- Presentation in the ER: Triage on arrival using the Manchester Triage System (MTS) [12], Glasgow Coma Scale (GCS) [13], compromises to clinical assessability, and intoxication, trauma CT during primary survey;
- Vital signs: Systolic blood pressure < 100 or >160 mmHg, heart rate < 50/min or >100/min, positive shock index, and peripheral oxygen saturation < 95%;
- Blood tests: leukocytes > 10.000/mL, hemoglobin < 12 g/dL, international normalized ratio (INR) < 0.85.

The following outcome variables were analyzed:

- Additional, not previously planned, radiological examinations;
- Additional, not previously planned, blood tests;
- Additional injuries sustained through the accident, not diagnosed during primary or secondary survey (“missed injuries”) [14];
- Additional non-traumatic diagnoses, not present in the admission report;
- Additional therapy, not previously planned (e.g., orthoses, casts);
- i.v. analgesia.

Outcomes were reported descriptively with absolute numbers and percentages. If any of these endpoints were reached, the stay was categorized as “eventful”. Two univariate case–control analyses were performed, one comparing patients with eventful and uneventful stay and one comparing patients with and without missed injury. Here, for continuous variables with group sizes *n* ≥ 30 a two-sided *t*-test, for ordinal variables and continuous variables with group sizes *n* < 30 a Mann–Whitney U test, and for categorical variables Fisher’s exact test were used. *p*-values < 0.05 were considered statistically significant. Data were analyzed using R (R Core Team (2023)). _R: A Language and Environment for Statistical Computing_. R Foundation for Statistical Computing, Vienna, Austria. https://R-project.org/, accessed on 1 November 2025).

Portions of this manuscript were supported by the use of ChatGPT (OpenAI, San Francisco, CA, USA; version GPT-5.1, accessed 12 December 2025). The tool was used for language editing and text structuring, and all content was checked and verified by the authors.

## 3. Results

### 3.1. Inclusion and Exclusion

From January 2022 to September 2023, a total of 6515 patients were admitted for inpatient treatment at the study site via the ER. After initial screening, 496 remained who were not admitted for severe injuries, inpatient medication, or social reasons, but solely based on accident mechanism. Eighty-five patients were excluded because they had only fallen from standing height, not meeting the criteria of HEA. Another 48 patients were <18 years and were therefore excluded, leaving 363 patients for the final analysis.

### 3.2. Baseline Characteristics

Mean age was 42.0 years (SD 17.8, range 18–87), 33.3% were female. Most had no significant comorbidities (mean age-adjusted CCI 0.8, CCI ≤ 1 in 77.7%, ASA ≤ 2 in 88.4%). DOAKs or APM were taken by 8.8%. For no patient or region, maximum AIS was >2, mean ISS was 2.4 (SD 1.5, range 0–9). Car accidents were most frequent (35.3%, *n* = 128), followed by falls < 3 m (15.4%, *n* = 56). Details are presented in the first column of Table 2.

Table 3, column 1 gives details on clinical presentation, vital signs and blood test results of patients upon admission to the ER. According to the MTS, 88.4% of patients were category “Orange” or “Yellow”. The vast majority of patients presented with normal GCS and vital signs, few had hypertension (15.2%) or tachycardia (11.3%). In blood tests, leukocytosis >10.000/mL was frequent (30.3%).

### 3.3. Events

Events during the hospital stay are first detailed (Table 4) and then analyzed regarding potential risk factors in case–control analyses (included in Table 2 and Table 3).

Out of 363 patients, 86.0% (*n* = 312) experienced an uneventful stay. For the remaining 14.0% (*n* = 51), additional, not initially planned measures were taken or additional diagnoses made during the stay (eventful stay, Table 4). Additional radiological examinations were most frequent (8.5%), followed by additional blood tests (6.9%). A new diagnosis was found in 6.3% and 6.1% received additional therapeutic interventions.

### 3.4. Missed Injuries and Management

Table 2 gave an overview of injury patterns according to AIS for distinct body regions based on diagnoses apparent upon admission. The following section first details initially missed injuries that only became apparent during the hospital stay (Table 5), then presents case–control analyses for potential risk factors (Table 5 and Table 6).

In 3.9% (*n* = 14) initially missed injuries were found during the hospital stay (Table 5). In two cases (0.6%), potentially life-threatening injuries were detected: One 60-year-old male patient after a motorcycle crash had initially received eFAST (extended focused assessment with sonography in trauma). Later, a chest x-ray revealed two rib fractures and a marginal pneumothorax, which had not been detected with eFAST. This patient, however, refused any further measures including placement of a chest tube and left the hospital against medical recommendation after 25 h of surveillance. He was not readmitted to our site and his outcome is unknown. An 18-year-old male patient after a car accident had initially received eFAST and a whole-body CT-scan. Within six hours of surveillance, he developed an acute abdomen. In the following blood test he had falling hemoglobin levels and was transferred to general surgery for emergency laparotomy due to intraabdominal bleeding due to small intestine injury

### 3.5. Risk Factors for Events and Missed Injuries

In univariate analyses, patients with eventful stay (*n* = 51) and patients with uneventful stay (*n* = 312) were compared to identify potential predictors. Regarding demographics and injury pattern, there were no significant differences between the groups (Table 2). Apart from bicycle accidents, which were significantly more frequent in the group with eventful stay (31.3% vs. 15.4%, *p* = 0.009), none of the accident mechanisms was a significant predictor (Table 2). No significant predictors were found among the variables assessed during the examinations in the ER (Table 3).

For missed injuries, no significant predictors were identified among demographic variables, injury pattern, and accident mechanism (Table 6). None of the variables documented in the ER significantly predicted missed injuries (Table 7).

## 4. Discussion

This retrospective study of 363 patients without apparent severe injury admitted for inpatient surveillance after HEAs analyzed events, interventions, and new diagnoses during inpatient surveillance to evaluate its clinical utility and to identify risk factors for an eventful stay and missed injuries. Our findings largely confirm the low yield of this measure with 86% of patients experiencing an uneventful stay. In only 3.9%, missed injuries were detected before discharge. No robust predictors for missed injuries were identified, reflecting the low-risk and low-variability nature of the cohort. However, two patients (0.6%) were diagnosed with severe injuries that were initially not apparent, underlining the low but persistent risk of life-threatening injury in these patients.

### 4.1. Rates and Predictors of Missed Injuries

Previous studies on uninjured or only lightly injured patients also found a low yield of inpatient surveillance after HEAs [4,5,6]. Lansink et al. studied 503 patients with no more than two minor injuries after high-energy accidents in a bi-center retrospective study [4]. They found no complications or readmissions within 30 days after trauma and no missed injuries were reported, leading the authors to the conclusion that surveillance of these patients was unnecessary. As opposed to more current studies, including ours, certain exclusion criteria limited generalizability: Their exclusion of patients with coagulopathy, anticoagulant medication, or ischemic heart disease likely resulted in a lower-risk population with a younger mean age (30.5 years vs. 42.0 years in our cohort). The authors did not report specific interventions or events during the hospital stay.

Heinrich et al. analyzed 112 patients with AIS ≤ 1 and ISS ≤ 3 observed for at least 24 h after HEAs [5]. They noted no significant increase in AIS or ISS during the hospital stay. New diagnoses were found in 8.0%, but no interventions were necessary for any patient. However, while no definition of what constituted an intervention was given, additional diagnostic measures such as radiographs did occur in their cohort.

Van Aert et al. investigated 388 patients with AIS ≤ 2 admitted for observation after trauma and detected missed injuries in 3.4%, while serious damage was reportedly prevented in 0.5% of patients [6]. Higher ISS and alcohol intoxication upon admission were significant risk factors for missed injuries in univariate analyses. As opposed to our study, patients were also included if the reason for admission was insufficient home care or low-energy mechanisms such as falls from standing height, limiting comparability to our patient cohort. However, comparable rates of missed injuries and serious injuries were found.

### 4.2. Missed Injuries vs. Eventful Stay

Thus, across comparable studies, the low rates of missed injuries and the very small proportion of serious conditions detected during inpatient surveillance have led to the assumption that routine admission of patients without relevant injuries may be unnecessary. To expand the perspective on potential benefits of surveillance in this cohort, we applied a broader, process-oriented definition of an “eventful stay,” encompassing not only new diagnoses but also additional diagnostics, blood tests, therapeutic interventions, and analgesia—elements that have not previously been reported in detail. Using these criteria, 14.0% of patients in our cohort received additional in-hospital care. While some measures, such as standard radiographs, might theoretically have been performed in an outpatient setting, 6.1% of patients required therapeutic interventions, including casts, orthoses, or crutches, that would likely have been delayed outside the hospital. Thus, our findings demonstrate that even in the absence of serious injuries, the cumulative resource use associated with this patient group is non-negligible, and that a narrow focus on AIS/ISS progression or missed injuries underestimates the true scope of clinical work generated during inpatient observation.

### 4.3. Predictors of Missed Injuries and Eventful Stays

We did not identify reliable predictors of either an eventful stay or missed injuries. In the univariate analyses, bicycle accidents were significantly associated with an eventful stay. However, statistical fragility of this finding must be considered, since in spite of multiple testing, no adjustment of the significance level was applied in this exploratory study. Also, the category “bicycle accident” comprised in small subgroups of individuals with bike falls but also bike-vs.-car and bike-vs.-bike collisions, further emphasizing the importance of cautious interpretation. Still, particularly careful examinations of individuals after bicycle accidents seems warranted.

In the population of trauma patients with various severity, a number of risk factors for missed injuries were identified in the past, as outlined by a recent systematic review by Vieira et al. [2]. Among these are high injury severity and polytrauma, as well as altered mental status and otherwise impaired clinical examination [2,15]. These findings were partly reproduced by von Aert et al. [6] in their cohort of lightly injured patients, but could not be observed in our cohort or the studies by Heinrich et al. [5] and Lansink et al. [4]. Other factors identified by Vieira et al. are false-negative imaging studies and radiology misinterpretation, patient-related factors such as obesity, advanced age and pregnancy, and human factors such as inexperience, fatigue or cognitive bias of the treatment team [2]. The authors conclude that missed injuries are multifactorial, which is plausible and might also be the reason why identifying such risk factors in populations of lightly or uninjured patients is so difficult: The relative rarity of the endpoint “missed injury” in this cohort yields logistic regression impractical. Additionally, the absence of identifiable predictors for either eventful stay or missed injuries in our or in comparable studies must be interpreted in the context of the selected population. Most established risk factors, such as high injury severity, altered mental status, hemodynamic instability, or polytrauma, were absent by design. This low variability likely explains why previously reported predictors did not emerge. In such narrowly selected low-risk cohorts, the inherent rarity of the outcome further limits statistical power to detect meaningful associations.

Our findings add to existing evidence that HEA, although useful for initial triage, is a poor discriminator for clinically relevant injury once primary assessment and trauma imaging are normal. Previous studies demonstrate that while the mechanism of injury influences injury patterns [16,17], it cannot reliably predict which patients will develop clinically significant findings during observation. This underscores the limited utility of mechanism alone as an admission criterion in the absence of physiologic or anatomic abnormalities.

### 4.4. Residual Risk of Serious Injury

Although the vast majority of patients with normal initial evaluation after high-energy trauma do not develop clinically relevant findings, numerous studies highlight the persistent “residual risk” of serious injuries that become apparent only hours later. This phenomenon has been described in cohorts of CT-negative blunt abdominal trauma patients, in whom delayed intraabdominal injuries occur in 0.1–0.3% [3,18], and in broader trauma populations where hollow-viscus, vascular, or thoracic injuries may evolve despite initially unremarkable imaging [19]. Systematic reviews have emphasized that such cases reflect the inherent limitations of early diagnostics and the temporal evolution of certain injuries rather than failures of initial assessment [2]. Our rate of severe missed injuries, affecting one man with intraabdominal bleeding and one man with marginal pneumothorax (0.6%), lies within this range. The former case highlights the limitations of early imaging and the potentially misleading clinical presentation, particularly in younger patients, especially well. In this context, the mechanism of injury, such as motor vehicle accident, has been found to be an important predictor of intra-abdominal trauma and should be integrated into clinical decision-making [20].

### 4.5. Implications for Clinical Pathways

This illustrates the current dilemma of handling patients after HEAs with normal findings in the ER: Admission of all these patients is a large investment of resources in stressed healthcare systems all over the world, while completely abandoning surveillance may carry a small but non-negligible risk of severe patient harm. Since many severe injuries do manifest within the first hours after trauma [3,19], however, the traditional 24 h surveillance period may be replaceable by shorter stays in specialized observation units. For the detection of less severe injuries which may become apparent after the immediate post-traumatic period, structured outpatient reassessment within 24–48 h could be viable. A combined approach of short in-hospital observation followed by structured outpatient review may offer a safe and efficient alternative to 24 h inpatient surveillance in low-risk patients. This could be flanked with safety-net instructions, ensuring sufficient home care in case of discharge and informing patients and their social surroundings about red flags that should trigger immediate medical investigation.

### 4.6. Strengths and Limitations

Several limitations must be acknowledged. The study’s single-center and retrospective design limits generalizability, as admission practices and diagnostic resources may vary across institutions. Due to the retrospective design, a standardized operationalization of HEA criteria was not consistently possible. In particular, key parameters such as collision velocity or fall height were frequently based on patient-reported information and were uncertain in some cases. As a result, the classification of HEAs in this study reflects routine clinical decision-making rather than a strictly protocolized definition. Data on patients with HEAs who were discharged from the emergency department were not available, limiting the assessment of the overall patient population and selection process. Furthermore, some detailed information on accident mechanisms (e.g., collision type or impact direction) was not consistently available, as documentation was based on routine clinical records and initial patient-reported information, thereby limiting more granular analysis of injury patterns.

The absolute number of events, particularly severe missed injuries, was small, reducing statistical power. As a result, only univariate analyses were conducted; while this avoids overfitting given the low event rate, it does not allow exploration of interactions between variables or multivariable adjustment. We also did not differentiate between type I missed injuries (detected during the tertiary survey) and type II missed injuries (identified later during hospitalization), which may have masked differences in preventability or clinical relevance. Moreover, not all missed injuries necessarily represent clinically meaningful events, and the interpretation of their significance remains complex. Finally, patients under 18 years of age were excluded, limiting applicability to pediatric and adolescent trauma populations, in whom imaging thresholds and diagnostic strategies differ and merit separate evaluation. Among the variables possibly influencing missed injuries but not accounted for in our study were the experience of the attending physician and the number of patients in the ER.

As a strength, this study was conducted at a Level I trauma center with standardized trauma assessment pathways, including routine tertiary surveys, which increases the reliability of clinical documentation and injury detection. The inclusion criteria focused specifically on patients admitted solely on the basis of high-energy mechanism without apparent injury, allowing for targeted insights into a clinically relevant but understudied patient group. In contrast to previous work, we applied a broader outcome framework that went beyond missed injuries alone, capturing additional diagnostics, therapies, and unplanned clinical measures, thereby providing a more comprehensive picture of the true in-hospital resource utilization associated with this cohort.

### 4.7. Conclusions

In conclusion, the question of whether patients with normal clinical and radiologic findings after a HEA require routine inpatient surveillance remains unresolved. Our study demonstrates that while a subset of patients benefits from additional in-hospital diagnostics or treatment, the overall yield of mandatory admission is low, making its routine use problematic in the context of strained healthcare resources. At the same time, no validated tool currently exists to identify the small proportion of patients at risk for clinically significant injuries that are not apparent at initial assessment. In view of the ongoing shift toward ambulatory care pathways, hybrid models consisting of short-term observation during the first post-traumatic hours followed by structured outpatient reassessment may represent a promising and less resource-intensive alternative.

## Figures and Tables

**Table 1:** Inclusion and exclusion criteria.

**Category**	**Inclusion Criteria**
Mechanism of accident	Mechanism-based criteria oriented on S3 guideline B-criteria, including: ○ High-speed motor vehicle collision ○ Vehicle rollover or ejection ○ Pedestrian struck ○ Motorcycle/bicycle collision ○ Fall from height (>3 m or >2–3× body height) ○ Other high-energy mechanisms (e.g., crush injury, explosion)
Clinical selection	Admitted for in-hospital observation based on above criteria and trauma surgeon judgment
**Category**	**Exclusion criteria**
Age	<18 years
Severe injury	Injury requiring immediate inpatient surgical care Injury requiring immediate inpatient surveillance (e.g., massive swelling, ≥3 fractured ribs) Traumatic brain injury, including traumatic brain injury grade I
Inpatient medication	Conditions requiring i.v. antibiotics Conditions requiring i.v. analgesia
Social reasons for admission	Limited mobility Limited domestic support
Low-energy trauma	Fall from standing height

**Table 2:** Demographic baseline characteristics, injuries and accident mechanisms of the total cohort (*n* = 363) and univariate case–control analyses comparing patients with uneventful (*n* = 312) and eventful stays (*n* = 51).

Variable	Total Cohort (*n* = 363)		Uneventful Stay (*n* = 312)		Eventful Stay (*n* = 51)		*p*-Value
**Demographics**							
Age [mean (SD)]	42.0	(17.8)	42.3	(17.8)	40.3	(17.6)	0.443 ^1^
Sex							
female	121	33.3%	109	34.9%	12	23.5%	0.149 ^2^
male	242	66.7%	203	65.1%	39	76.5%	
CCI [mean (SD)]	0.8	(1.3)	0.8	(1.3)	0.8	(1.4)	0.518 ^3^
ASA > 2	42	11.6%	37	11.9%	5	9.8%	0.816 ^2^
DOAK/APM	32	8.8%	27	8.7%	5	9.8%	0.79 ^2^
**Injury**							
ISS [mean (SD)]	2.4	(1.5)	2.4	(1.5)	2.6	(1.6)	0.668 ^3^
AIS Head > 0	109	30.0%	97	31.1%	12	23.5%	0.325 ^2^
AIS Face > 0	41	11.3%	32	10.3%	9	17.6%	0.149 ^2^
AIS Neck > 0	2	0.6%	2	0.6%	0	0.0%	1 ^2^
AIS Thorax > 0	105	28.9%	94	30.1%	11	21.6%	0.246 ^2^
AIS Abdomen > 0	18	5.0%	15	4.8%	3	5.9%	0.727 ^2^
AIS Spine > 0	127	35.0%	107	34.3%	20	39.2%	0.528 ^2^
AIS Upper Extremities > 0	169	46.6%	141	45.2%	28	54.9%	0.227 ^2^
AIS Lower Extremities > 0	154	42.4%	137	43.9%	17	33.3%	0.172 ^2^
AIS Other > 0	2	0.6%	2	0.6%	0	0.0%	1 ^2^
**Accident**							
Car	128	35.3%	113	36.2%	15	29.4%	0.430 ^2^
Bicycle	64	17.6%	48	15.4%	16	31.3%	**0.009** ^2^
Motorcycle	61	16.8%	51	16.3%	10	19.6%	0.548 ^2^
Fall < 3 m	56	15.4%	53	17.0%	3	5.9%	0.057 ^2^
Fall > 3 m	12	3.3%	10	3.2%	2	3.9%	0.679 ^2^
other	42	11.5%	37	11.8%	5	9.8%	0.816 ^2^

Statistical tests used: ^1^ Two-sided *t*-test, ^2^ Fisher’s exact test, ^3^ Mann–Whitney U test. Bold denotes statistical significance. Abbreviations: AIS, abbreviated injury scale; APM, anti-platelet medication; ASA, American Society of Anesthesiologists; CCI, age-adjusted Charlson Comorbidity Index; DOAKs, direct oral anticoagulants; SD, standard deviation.

**Table 3:** Baseline characteristics of presentation, vital signs and blood tests upon admission in the total cohort (*n* = 363) and univariate case–control analyses comparing patients with uneventful (*n* = 312) and eventful stay (*n* = 51).

Variable	Total Cohort (*n* = 363)		Uneventful Stay (*n* = 312)		Eventful Stay (*n* = 51)		*p*-Value
**Presentation**							
MTS category							0.540 ^2^
Red	2	0.6%	2	0.6%	0	0.0%	
Orange	119	32.8%	100	32.1%	19	37.3%	
Yellow	202	55.6%	175	56.1%	27	52.9%	
Green	40	11.0%	35	11.2%	5	9.8%	
Blue	0	0.0%	0	0.0%	0	0.0%	
GCS [mean (SD)]	15.0	(0.2)	15.0	(0.2)	15.0	(0.0)	0.320 ^2^
Clinical assessability compromised	46	12.7%	43	13.8%	3	5.9%	0.170 ^1^
Intoxication	30	8.3%	29	9.3%	1	2.0%	0.099 ^1^
Trauma-CT Scan	194	53.4%	168	53.8%	26	51.0%	0.763 ^1^
**Vital signs**							
BP syst < 100 mmHg	2	0.6%	2	0.6%	0	0.0%	1 ^1^
BP syst > 160 mmHg	55	15.2%	50	16.0%	5	9.8%	0.298 ^1^
HR < 50/min	1	0.3%	1	0.3%	0	0.0%	1 ^1^
HR > 100/min	41	11.3%	35	11.2%	6	11.8%	0.816 ^1^
Shock Index positive	1	0.3%	1	0.3%	0	0.0%	1 ^2^
SpO_2_ < 95%	15	4.1%	12	3.8%	3	5.9%	0.452 ^1^
**Blood tests**							
Leukocytes > 10.000/mL	110	30.3%	95	30.4%	15	29.4%	1 ^1^
Hb < 12 g/dL	23	6.3%	20	6.4%	3	5.9%	1 ^1^
INR < 0.85	3	0.8%	3	1.0%	0	0.0%	1 ^1^

Statistical tests used: ^1^ Fisher’s exact test, ^2^ Mann–Whitney U test. Abbreviations: BP, blood pressure; GCS, Glasgow Coma Scale; Hb, hemoglobin; HR, heart rate; INR, international normalized ratio; MTS, Manchester triage system; SD, standard deviation; SpO_2_, peripheral oxygen saturation.

**Table 4:** Events and interventions during the hospital stay. Total cohort *n* = 363.

Event	*n*	%
**Any event**	**51**	**14.0%**
**Additional radiological diagnostics**	**31**	**8.5%**
CT cranium and cervical spine	4	1.1%
CT abdomen	1	0.3%
CT extremities	3	0.8%
MRI spine	3	0.8%
MRI extremities	1	0.3%
X-ray thorax	3	0.8%
X-ray spine	3	0.8%
X-ray extremities	13	3.6%
**Additional blood test**	**25**	**6.9%**
**New diagnosis**	**24**	**6.6%**
Missed injury	14	3.9%
Severe Injury	2	0.6%
Non-traumatic diagnosis	10	2.8%
**Additional therapy**	**22**	**6.1%**
Orthosis/cast	11	3.0%
Prolonged surveillance	3	0.8%
I.v. analgesia	2	0.6%
Crutches	1	0.3%
Emergency laparotomy	1	0.3%
Chest tube *	1	0.3%
Other	3	0.8%

* Chest tube was recommended because of pneumothrax, however patient refused.

**Table 5:** Missed injuries (*n* = 14) and management.

Age	Gender	Accident	Missed Injury	Management
44	m	Bike	Cervical plexus lesion	Outpatient MRI and EMG
62	m	Car	LFTA lesion	Ankle orthosis, conservative
62	m	Fall < 3 m	Lumbar vertebra 1 fracture	Conservative
33	f	Motorcycle	Tibiofibular syndesmotic lesion	Ankle splint, outpatient MRI
24	m	Motorcycle	Proximal phalanx fracture digit 5 hand	Cast, outpatient surgery
53	m	Car	AC joint injury	Shoulder orthosis, outpatient surgery
20	m	Car	Scaphoid fracture	Cast, conservative
26	m	Car	Partial ACL re-rupture	Crutches, orthosis, conservative
18	m	Car	Intraabdominal bleeding due to small intestine injury	Emergency laparotomy
40	f	Bike	Tuberculum majus fracture	Shoulder orthosis, outpatient MRI
60	m	Motorcycle	Pneumothorax, rib fracture 5 and 6	Chest tube recommended, self-discharge against medical advice
32	m	Car	Radial head fracture	Orthosis, conservative
19	m	E-Scooter	AC joint injury	Shoulder orthosis, outpatient surgery
27	m	Car	Distal phalanx fracture digit 1 foot	Foot orthosis, conservative

Abbreviations: AC, acromioclavicular; ACL, anterior cruciate ligament; EMG, electromyogramm; LFTA, ligamentum fibulotalare anterius.

**Table 6:** Demographic baseline characteristics, injuries and accident mechanisms of the total cohort (*n* = 363) and univariate case–control analyses comparing patients without missed injury (*n* = 349) and with missed injury (*n* = 14).

Variable	Total Cohort (*n* = 363)		No Missed Injury (*n* = 349)		Missed Injury (*n* = 14)		*p*-Value
**Demographics**							
Age [mean (SD)]	42.0	(17.8)	42.2	(17.8)	37.1	(16.4)	0.304 ^2^
Sex							
female	121	33.3%	119	34.1%	2	14.3%	0.155 ^1^
male	242	66.7%	230	65.9%	12	85.7%	
CCI [mean (SD)]	0.8	(1.3)	0.8	(1.3)	0.5	(0.9)	0.406 ^2^
ASA > 2	42	11.6%	41	11.7%	1	7.1%	1 ^1^
DOAK/APM	32	8.8%	31	8.9%	1	7.1%	1 ^1^
**Injury**							
ISS [mean (SD)]	2.4	(1.5)	2.4	(1.5)	2.8	(1.4)	0.293 ^1^
AIS Head > 0	109	30.0%	107	30.7%	2	14.3%	0.245 ^1^
AIS Face > 0	41	11.3%	39	11.2%	2	14.3%	0.664 ^1^
AIS Neck > 0	2	0.6%	2	0.6%	0	0.0%	1 ^1^
AIS Thorax > 0	105	28.9%	99	28.4%	6	42.9%	0.242 ^1^
AIS Abdomen > 0	18	5.0%	16	4.6%	2	14.3%	0.148 ^1^
AIS Spine > 0	127	35.0%	122	35.0%	5	35.7%	1 ^1^
AIS Upper Extremities > 0	169	46.6%	160	45.8%	9	64.3%	0.274 ^1^
AIS Lower Extremities > 0	154	42.4%	149	42.7%	5	35.7%	0.784 ^1^
AIS Other > 0	2	0.6%	2	0.6%	0	0.0%	1 ^1^
**Accident**							
Car	128	35.3%	126	36.1%	2	14.3%	0.151 ^1^
Bicycle	64	17.6%	60	17.2%	4	28.6%	0.283 ^1^
Motorcycle	61	16.8%	56	16.0%	5	35.7%	0.067 ^1^
Fall < 3 m	56	15.4%	55	15.8%	1	7.1%	0.705 ^1^
Fall > 3 m	12	3.3%	12	3.4%	0	0.0%	1 ^1^
other	42	11.6%	40	11.5%	2	14.3%	0.670 ^1^

Statistical tests used: ^1^ Fisher’s exact test, ^2^ Mann–Whitney U test. Abbreviations: AIS, abbreviated injury scale; APM, anti-platelet medication; ASA, American Society of Anesthesiologists; CCI, age-adjusted Charlson Comorbidity Index; DOAKs, direct oral anticoagulants; SD, standard deviation.

**Table 7:** Baseline characteristics of presentation, vital signs and blood tests upon admission in the total cohort (*n* = 363) and univariate case–control analyses comparing patients without missed injury (*n* = 349) and with missed injury (*n* = 14).

Variable	Total Cohort (*n* = 363)		No Missed Injury (*n* = 349)		Missed Injury (*n* = 14)		*p*-Value
**Presentation**							
MTS category							0.423 ^2^
Red	2	0.6%	2	0.6%	0	0.0%	
Orange	119	32.8%	118	33.8%	1	7.1%	
Yellow	202	55.6%	195	55.9%	7	50.0%	
Green	40	11.0%	34	9.7%	6	42.9%	
Blue	0	0.0%	0	0.0%	0	0.0%	
GCS [mean (SD)]	15.0	(0.2)	15.0	(0.2)	15.0	(0.0)	0.623 ^2^
Clinical assessability compromised	46	12.7%	46	13.2%	0	0.0%	0.231 ^1^
Intoxication	30	8.3%	30	8.6%	0	0.0%	0.617 ^1^
Trauma-CT Scan	194	53.4%	185	53.0%	9	64.3%	0.586 ^1^
**Vital signs**							
BP syst < 100 mmHg	2	0.6%	2	0.6%	0	0.0%	1 ^1^
BP syst > 160 mmHg	55	15.2%	54	15.5%	1	7.1%	0.704 ^1^
HR < 50/min	1	0.3%	1	0.3%	0	0.0%	1 ^1^
HR > 100/min	41	11.3%	39	11.2%	2	14.3%	0.664 ^1^
Shock Index positive	1	0.3%	1	0.3%	0	0.0%	1 ^1^
SpO_2_ < 95%	15	4.1%	14	4.0%	1	7.1%	0.452 ^1^
**Blood** **tests**							
Leukocytes > 10.000/mL	110	30.3%	104	29.8%	6	42.9%	0.383 ^1^
Hb < 12 g/dL	23	6.3%	23	6.6%	0	0.0%	0.611 ^1^
INR < 0.85	3	0.8%	3	0.9%	0	0.0%	1 ^1^

Statistical tests used: ^1^ Fisher’s exact test, ^2^ Mann–Whitney U test. Abbreviations: BP, blood pressure; GCS, Glasgow Coma Scale; Hb, hemoglobin; HR, heart rate; INR, international normalized ratio; MTS, Manchester triage system; SD, standard deviation; SpO_2_, peripheral oxygen saturation.

## Data Availability

Data can be provided by the authors upon reasonable request.

## Abbreviations

The following abbreviations are used in this manuscript:

AIS	Abbreviated Injury Scale
APM	Anti-platelet medication
ASA	American Society of Anesthesiologists
ATLS^®^	Advanced Trauma Life Support^®^
CCI	Charlson Comorbidity Index
DOAKs	Direct oral anticoagulants
ER	Emergency room
GCS	Glasgow Coma Scale
HEA	High-energy accident
INR	International normalized ratio
MTS	Manchester Triage System
SD	Standard deviation
